# Beneficial Impact of an Extract from the Berries of *Aronia melanocarpa* L. on the Oxidative-Reductive Status of the Submandibular Gland of Rats Exposed to Cadmium

**DOI:** 10.3390/antiox9020185

**Published:** 2020-02-22

**Authors:** Adam Dąbrowski, Barbara M. Onopiuk, Halina Car, Paweł Onopiuk, Zofia N. Dąbrowska, Joanna Rogalska, Małgorzata M. Brzóska, Ewa Dąbrowska

**Affiliations:** 1 Private Dental Office in Bialystok, Rzemieślnicza 37, 15-773 Bialystok, Poland; bnm2@wp.pl (A.D.);; 2 Department of Clinical Pharmacology, Medical University of Bialystok, Jerzego Waszyngtona 15A, 15-174 Bialystok, Poland; hcar@umb.edu.pl; 3 Department of Otolaryngology, Medical University of Bialystok, Marii Skłodowskiej- Curie 24A, 15-276 Bialystok, Poland; pawel32167@gmail.com; 4 Department of Periodontal and Oral Mucosa Diseases, Medical University of Bialystok, Marii Skłodowskiej-Curie 24A, 15-276 Bialystok, Poland; 5 Department of Toxicology, Medical University of Bialystok, Adama Mickiewicza 2C, 15-222 Bialystok, Poland; joanna.rogalska@umb.edu.pl (J.R.); malgorzata.brzoska@umb.edu.pl (M.M.B.); 6 Department of Gerostomatology, Medical University of Bialystok, Akademicka 3, 15-286 Bialystok, Poland; helpdent@umb.edu.pl

**Keywords:** *Aronia melanocarpa* berries extract, polyphenols, cadmium, submandibular gland, salivary gland, oxidative/antioxidative status, oxidative-reductive status, oxidative stress, lipid peroxidation, rats

## Abstract

Oxidative stress underlies the pathomechanisms of toxic action of cadmium (Cd), including its damaging impact on the oral cavity. This study investigated whether the administration of an extract from *Aronia melanocarpa* L. berries (AME), characterized by their strong antioxidative potential, may have a beneficial impact on the oxidative-reductive status of the submandibular gland in an experimental model of low-level and moderate human environmental exposure to cadmium. The main markers of the antioxidative status (glutathione reductase, superoxide dismutase, catalase, reduced glutathione, total antioxidative status (TAS)), total oxidative status (TOS), oxidative stress index (OSI = TOS/TAS), and lipid peroxides, as well as cadmium concentration, were evaluated in the submandibular gland tissue of female Wistar rats who received a 0.1% aqueous AME and/or a diet containing 0, 1, and 5 mg Cd/kg for 3 and 10 months. The treatment with cadmium decreased the activities of antioxidative enzymes (29%–74%), reduced glutathione concentration (45%–52%), and TAS and increased TOS, resulting in the development of oxidative stress and enhanced concentration of lipid peroxides in the submandibular gland. The administration of AME at both levels of exposure to cadmium offered significant protection against these actions of this xenobiotic. After the 10 month exposure to the 1 and 5 mg Cd/kg diet, TAS was decreased by 77% and 83%, respectively, TOS, OSI, and lipid peroxides concentration were increased by 50% and 52%, respectively, 11.8-fold and 14.4-fold, respectively, and 2.3-fold and 4.3-fold, respectively, whereas, in the case of the extract co-administration, the values of these parameters did not differ compared to the control group. The results indicate that the consumption of aronia products under exposure to cadmium may have a beneficial impact on the oxidative-reductive status of the submandibular gland and prevent oxidative stress development and enhanced lipid peroxidation in this salivary gland.

## 1. Introduction

It is well known that oxidative stress underlies the pathomechanisms of the development of various diseases, including diseases of the oral cavity [1,2], as well as of the toxic action of numerous xenobiotics [3,4,5,6]. Cadmium (Cd) is one of them [3,6]. The growing industrial use of this element in the last decades has resulted in the contamination of the natural environment with this xenobiotic, and unavoidable human exposure to it during a lifetime [7,8,9]. Nowadays, this heavy metal belongs to the main environmental contaminants in economically developed countries [3,7,9]. Cadmium is a pollutant of air, water, and soil, as thus it is present in food, which is the main source of the general population’s exposure to this heavy metal [9,10,11]. Furthermore, an additional source of intoxication with this xenobiotic is tobacco smoking [8,12]. Cadmium concentrations in the blood and urine of habitual smokers are markedly higher than in non-smokers [8,12,13]. 

The harmfulness of cadmium to the human’s and animals’ organism is well known and widely reported [3,9,11]. This heavy metal is characterized by strong cumulative properties, and thus its content in the body increases with the duration of exposure and may be a cause of damage to various organs and systems [3,14]. Recent epidemiological studies provide more and more evidence that environmental exposure to this xenobiotic, nowadays occurring in economically developed countries, creates a threat to the health of the general population, mainly including a risk of damage to the kidney, liver, cardiovascular system, and skeleton, as well as the development of cancer and the deterioration of cognitive functions such as hearing and vision [3,9,13,15,16,17,18]. It has been revealed that numerous effects of toxic action of cadmium, including the damaging impact on the organs of the oral cavity, result from its pro-oxidative properties [5,14,19,20,21,22,23]. This xenobiotic indirectly mediates the generation of free radicals (FR) and reactive oxygen species (ROS) by weakening the antioxidative barrier (enzymatic and non-enzymatic), induction of the activity of oxidases, and damage to the mitochondria [3,14,21,22].

Because current environmental exposure to cadmium creates a threat to the health of the general population [3,8,10,11,13,15,16,17,18] and the lifetime human exposure to this xenobiotic will increase [9,10,11], the attention of researchers in recent years has been focused on finding effective ways to prevent the unfavourable health effects of exposure to this heavy metal. Taking into account the strong pro-oxidative properties of cadmium and the involvement of oxidative stress in the mechanisms of its damaging impact on various organs and systems [3,5,14,19,20,21,22,23], the greatest interest among the possible protective agents has been paid to natural products characterized by strong antioxidative properties, including polyphenol-rich ones [14,24,25,26].

One natural product possessing high antioxidative potential are the berries of *Aronia melanocarpa* L. (*A. melanocarpa,* (*Michx.*) Elliott, *Rosaceae*; black chokeberry), which are one of the richest sources of polyphenols (719–6902 mg/100 g) [24,27]. The antioxidative abilities of chokeberries result from properties of their ingredients such as proanthocyanidins, anthocyanins, flavonols, phenolic acids, and tannins, as well as vitamins and minerals [24,27]. The antioxidative potential of polyphenols is determined by their structure, the number and distribution of hydroxyl groups (–OH groups) in the aromatic ring, and the presence or absence of double bonds [27,28]. These compounds’ antioxidative action consists of a direct reaction with FR and their binding through the stabilization or delocalization of unpaired electrons, reductive properties (releasing electrons or hydrogen atoms), as well as increasing the dismutation of FR into compounds of significantly lower reactivity, and catalysing the transformation of FR into neutral products. Polyphenolic compounds inhibit the activity of a number of enzymes responsible for the production of ROS, including xanthine oxidase or myeloperoxidase and increase the activity of antioxidants (e.g., fat-soluble vitamins) and improve the total antioxidative potential. Moreover, due to the presence of an –OH group on the C-ring, they chelate metallic ions, e.g., iron and copper, which serve as active inductors of ROS [22,27,29]. Owing to the rich chemical composition, chokeberry fruits and their preparations show a wide spectrum of pro-health effects and there is a lot of evidence of their effective use in the prevention of civilization diseases, including atherosclerosis, diabetes, osteoporosis, and cancer [24,27,28].

Taking into account the strong antioxidative properties of chokeberries [24,27,28] and pro-oxidative action of cadmium [3,14,19,20], our research team has undertaken a comprehensive study, in the experimental model of low-level and moderate environmental human exposure to this xenobiotic (1 and 5 mg Cd/kg diet, respectively), to investigate whether the administration of an extract from the berries of *A. melanocarpa* (AME) may protect against this heavy metal toxicity. We have previously reported that the co-administration of 0.1% AME during the treatment with cadmium decreased the accumulation of this element in the body [30] and protected from its damaging impact on the skeleton [21,31] and liver [22,23], as well as improving the body status of zinc and copper [32]. In the case of the general population’s exposure to cadmium, via both diet and tobacco smoke, the oral cavity is the first place of the possible unfavourable action of this element. That is why, taking into account the findings that cadmium, due to the induction of oxidative stress, may have injurious action on the organs of the oral cavity, including the salivary glands [19,20,33], we have recognized it as necessary to estimate whether the administration of AME may also protect from this impact. 

With regard to the results of our research to date on the protective impact of AME towards cadmium-induced oxidative stress and its consequences [21,22,23], we have hypothesized that this extract may also protect from the pro-oxidative action of cadmium in the organs of the oral cavity, including the salivary glands. The aim of the present study was to investigate this hypothesis regarding the submandibular gland. For this purpose, the impact of the AME on the oxidative-reductive status of this salivary gland was estimated in the experimental model created by us of low-level and moderate human environmental exposure to cadmium. The main markers of the enzymatic (glutathione peroxidase (GPx), superoxide dismutase (SOD), and catalase (CAT)) and non-enzymatic (reduced glutathione (GSH)) antioxidative barrier and total antioxidative status (TAS), as well as total oxidative status (TOS), oxidative stress index (OSI), and lipid peroxides (LPO; a marker of lipid peroxidation), were evaluated in the submandibular gland tissue. Moreover, to assess the relationship between cadmium accumulation in this salivary gland and its pro-oxidative action, the concentration of this xenobiotic in the submandibular gland tissue was determined as well. Such a planned study allowed to investigate not only whether AME may protect from the pro-oxidative action of cadmium on the submandibular gland, but also to explain whether this impact is related to the extract’s antioxidative properties and its influence on cadmium accumulation in this salivary gland. The influence of the polyphenols and products abundant in these compounds on the salivary glands under exposure to cadmium has not been investigated until now. Our studies are the first regarding the effect of the extract from chokeberries on the oxidative-reductive status of the salivary glands at exposure to cadmium, and the present article is the first report presenting the impact on the submandibular gland.

## 2. Materials and Methods 

### 2.1. Chemicals

Cadmium chloride 2.5-hydrate (CdCl_2_ × 2.5 H_2_O), sodium chloride, potassium dihydrogen phosphate, and dipotassium hydrogen phosphate were purchased from POCh (Gliwice, Poland). Morbital and butyl-hydroxytoluene were received from Biowet (Pulawy, Poland) and Sigma-Aldrich Gmbh (Steinheim, Germany), respectively. Acetonitrile and trace-pure concentrated (65%) nitric acid were provided by Merck (Darmstadt, Germany). The kits for the determination of SOD (Superoxide Dismutase Assay Kit) and GSH (Glutathione Assay Kit) were received from Cayman (Ann Arbor, USA), whereas GPx and LPO were assayed with the use of BIOXYTECH GPx-340 and BIOXYTECH LPO-586 kits by Percipio Biosciences (Burlingame, CA, USA). The diagnostic ImAnOx (TAS) Kit and PerOx (TOS) Kit by Immundiagostik AG (Bensheim, Germany) were used for the determination of TAS and TOS, respectively. Hydrogen peroxide (30%), used for CAT determination, was purchased from CHEMPUR (Piekary Śląskie, Poland). Protein concentration was assayed using the BioMaxima kit (Lublin, Poland). A stock of standard solution of cadmium (1000 mg/L; Sigma-Aldrich, Buchs, Switzerland) and palladium matrix modifier (10 g/L; Merck) assigned for atomic absorption spectrometry (AAS method) were used. To check the analytical quality of cadmium determination, the Certified Reference Bovine Muscle (ERM-BB184, Geel, Belgium) was used. All the chemical reagents were characterized by a degree of purity for analysis, except for the reagents used under cadmium assay, which were assigned for trace analysis. Ultra-pure water, received from two-way water purification MAXIMA system (ELGA; Bucks, Great Britain), was used in all the measurements.

### 2.2. A. melanocarpa L. Extract and Diets Containing Cadmium

A lyophilized powdered extract from *A. melanocarpa* berries was received from Adamed Consumer Healthcare (Tuszyn, Poland). According to the producer (Certificate KJ 4/2010; Batch No. M100703), the extract contained 65.74% of polyphenols (including 18.65% of anthocyanins). The content of these compounds determined by us reached 61.24% ± 0.33% (mean ± standard error (SE)), including 20.23% ± 0.13% of anthocyanins (cyanidin 3-O-β-galactoside—8.01% ± 0.10%, cyanidin 3-O-α-arabinoside—3.32% ± 0.001%, cyanidin 3-O-β-glucoside—0.37% ± 0.001%). Moreover, the following polyphenolic compounds were quantified by us in the extract: total proanthocyanidins (12.99% ± 0.11%), total phenolic acids (11.09% ± 0.09%), chlorogenic acid (6.83% ± 0.01%), and total flavonoids (2.19% ± 0.1%) [31]. According to the producer, the extract also contains other components such as carotenoids, sugar, pectins, sugar alcohols (sorbitol, parasorboside), phytosterols, triterpenes, vitamins, and minerals.

The AME was administered to animals in the form of 0.1% aqueous solution prepared daily by dissolving the appropriate amount of the powdered extract in an appropriate volume of redistilled water (1 g of the extract per 1 L of redistilled water).

The diets containing cadmium at concentrations of 1 and 5 mg/kg were produced by Label Food ‘Morawski’ (Kcynia, Poland) by the addition of an appropriate amount of CdCl_2_ × 2.5 H_2_O into the components of the Labofeed H and Labofeed B diets. During the first 3 months of the experiment, the Labofeed H diet (breeding diet) was administered, and thereafter the Labofeed B diet (maintenance diet) was used. Cadmium concentration in the diets was confirmed by the manufacturer certificate and it was also quantified by us (1.09 ± 0.13 and 4.92 ± 0.53 mg Cd/kg; mean ± standard deviation – SD [30]). The concentration of this toxic element, determined by us in the standard Labofeed diets (H and B diets), reached 0.0584 ± 0.0049 mg/kg [30].

### 2.3. Animals

The experiment was performed on 96 young (3–4 weeks old and weighing about 50 g at baseline) female Wistar rats [Crl: WI (Han)] provided by the Laboratory Animal House in Brwinów (Poland; certified breeding). Throughout the experiment, the rats were kept in stainless steel cages (four animals in each) in standard conditions (temperature 22 ± 2 °C, relative humidity 50 ±10%, 12/12 h light-dark cycle). The animals had unlimited access to drinking water and food, the consumption of which was monitored throughout the study. 

### 2.4. Experimental Design

The submandibular salivary glands used in the current study were collected and secured during an experiment conducted at the Department of Toxicology at the Medical University of Bialystok (Poland), which was approved by the Local Ethics Committee for Animal Experiments in Bialystok (approval number 60/2009). The experimental model was described in detail previously [21,22,23,30,31,32,34]. 

The animals were randomized into the following six experimental groups of 16 rats each:

- Control group—the rats throughout the experiment (3 or 10 months) received redistilled water (containing < 0.05 μg Cd/L [30]) and the standard Labofeed fodder;
- AME group—the rats received as the only drinking fluid a 0.1% aqueous AME and the standard Labofeed fodder;
- Cd_1_ group—the rats throughout the experiment (3 or 10 months) received the Labofeed fodder containing 1 mg Cd/kg and redistilled water for drinking;
- Cd_1_ + AME group—during the whole period of maintaining on the diet containing 1 mg Cd/kg (3 or 10 months), the rats received the 0.1% aqueous AME as the only drinking fluid;
- Cd_5_ group—the rats received fodder containing 5 mg Cd/kg for 3 or 10 months and redistilled water for drinking;
- Cd_5_ + AME group—during the feeding with the diet containing 5 mg Cd/kg (3 or 10 months), the rats received the 0.1% aqueous AME as the only drinking fluid.

Throughout the experiment, no differences in the consumption of drinking water and food, as well as body weight gain, were noted among the experimental groups [30]. Moreover, there were no symptoms of abnormalities in the health status of the rats [30] and differences in the mean intake of AME and cadmium at particular timepoints (expressed in calculation per kilogram of body weight (kg b.w.)) throughout the study, regardless of whether they were administered separately or together (Table 1) [30,32]. The fact that cadmium concentrations in the blood and urine (markers of exposure to this heavy metal) of the animals maintained on the diets containing 1 and 5 mg Cd/kg alone and in combination with AME (0.103–0.306 μg/L and 0.0852–0.2558 μg/g creatinine, respectively, and 0.735–1.122 μg/L and 0.2839–0.6949 μg/g creatinine, respectively) [30] were within the ranges of values nowadays detected in the general population in economically developed countries [13,17,18] confirms that the experimental model reflects current environmental exposure to this xenobiotic.

After 3 months of the experiment, eight rats of each group (two cages with four animals each) were sacrificed, whereas all remaining animals were necropsied after 10 months. The animals (after overnight deprivation of food) were subjected to necropsy under barbiturate anaesthesia with Morbital (administered intraperitoneally in a dose of 30 mg/kg b.w.) and, after the blood collection (by cardiac puncture) and macroscopic examination, both submandibular glands (the left and the right one) were dissected (after previous separation of the sublingual salivary gland from the connective tissue bag). Immediately after collection, the salivary glands were rinsed thoroughly in ice-cold 0.9% sodium chloride (physiological saline) and gently dried on filter paper. Next, they were weighed with an automatic balance (OHAUS^®^, Nanikon, Switzerland; accuracy to 0.0001 g). All submandibular glands showed proper macroscopic picture and there were no differences in their weight (each gland weighted about 0.2 g) among the experimental groups.

### 2.5. Determination of Markers of the Oxidative/Antioxidative Status

In order to perform the planned assays of markers of the oxidative/antioxidative status, 20% homogenates of the submandibular gland tissue were prepared. The right submandibular gland and half of the left one (of known weight) were homogenized in cold 50 mM potassium phosphate buffer (pH = 7.4) with the use of a high-performance homogenizer (Ultra-Turrax T25; IKA, Staufen, Germany). In order to prevent autoxidation of the salivary gland tissue, 0.01 cm^3^ of 0.5 M butyl-hydroxytoluene in acetonitrile was added for each cm^3^ of the homogenate. The prepared homogenates were divided into two portions. One portion was centrifuged (MPW-350R centrifugator, Medical Instruments, Warsaw, Poland) at 700 × *g* for 20 minutes in 4 °C and the separated aliquot was used to the assay of CAT, GSH, TAS, TOS, and LPO. The second one was centrifuged at 20,000 × *g* for 30 minutes in 4 °C and the received aliquot was subjected to GPx and SOD assay [35]. The supernatants were stored in deep freezing (−80 °C) until the planned assays were performed. 

The activities of GPx and SOD and the concentrations of GSH and LPO were assayed with the use of commercially available kits (BIOXYTECH GPx-340 Assay, Superoxide Dismutase Assay Kit, Glutathione Assay Kit, and BIOXYTECH LPO-586 Assay). The precision of these measurements, expressed as a coefficient of variation (CV), was < 4.5%, 5%, 3.5%, and 6%, respectively. The activity of CAT was determined by the spectrophometric method according to Aebi [36] and the CV was < 5.3%. 

TAS and TOS were measured using the ImAnOx (TAS) Kit and PerOx (TOS) Kit. The values of TAS determined in the control samples included in the kit reached 195.3 ± 12.3 and 306.4 ± 17.5 µmol/L (mean ± SD, n = 2) and were within the manufacturer’s range of values (170–230 and 258-350 µmol/L, respectively). Similarly, the values of TOS assayed in the control samples included in the kit (145.9 ± 2.9 and 423.1 ± 6.8 µmol/L; mean ± SD, n = 2) were within the ranges of values stated by the producer (108–200 and 305–509 µmol/L, respectively). The precision of TAS and TOS assay (CV) was < 6% and < 2%. The value of OSI was mathematically calculated as the ratio of TOS and TAS.

The measured parameters of the oxidative-reductive status were adjusted for protein concentration. The assay of total protein was performed with the BioMaxima Kit (Lublin, Poland) according to the manufacturer’s instruction. 

All the above-mentioned assays performed with the use of commercial kits were conducted according to the producers’ instructions (describing also the rules of particular assays). The measurements were done using the spectrometer UV VIS SPECORD 50 PLUS (Analytik Jena, Jena, Germany) or MULTISCAN GO microplate reader (Thermo Scientific, Vantaa, Finland). Moreover, an automatic Wellwash 4 washer for microplates (Thermo Labsystems, Helsinki, Finland) was used. 

### 2.6. Determination of Cadmium Concentration

Known-weight halves of the left submandibular glands were wet-digested with a trace-pure concentrated nitric acid using UniClever II microwave system (Plazmatronika, Wroclaw, Poland) and then the wet-digests were diluted with ultra-pure water. The concentration of cadmium in such preparations was determined by the graphite furnace AAS method (GF AAS) using HITACHI Z-5000 atomic absorption spectrophotometer (Tokio, Japan) equipped with a graphite cuvette (Pyro cuvette A, Hitachi) and a hollow cathode lamp for this element assay (Photron, Narre Waren, Australia). The concentration of cadmium measured in the simultaneously analysed reference material (0.0020 ± 0.0001; Bovine muscle, ERM-BB184) was consistent with the value provided by the producer (0.0022 μg/g; uncertainty 0.0004 μg/g). The CV was < 6%. 

### 2.7. Statistical Analysis

The obtained results were analysed statistically using Statistica 10 software (StatSoft; Tulsa, OK, USA). The data are presented in figures in the form of mean ± SE for eight rats in each experimental group. In order to assess the statistical significance of differences between the experimental groups, the nonparametric Kruskal–Wallis test was performed. In cases when statistically significant differences occurred among the six experimental groups (level of statistical significance *p* < 0.05), the Kruskal–Wallis post hoc test was performed to compare individual groups and determine which two means differed statistically significantly. The possible impact of AME administration under exposure to cadmium on the values of estimated parameters was evaluated based on the statistical analysis of differences between the Cd_1_ + AME group or Cd_5_ + AME group and the respective group treated with cadmium alone (Cd_1_ group and Cd_5_ group, respectively), as well as differences between the groups co-administered with cadmium and AME (Cd_1_ + AME or Cd_5_ + AME groups) and the respective control group.

When the Kruskal–Wallis test revealed any influence of the co-administration of cadmium and AME on the investigated parameter, a two-way analysis of variance (ANOVA/MANOVA, test *F*) was conducted with the aim to discern the possible independent and/or interactive impact of these agents on this parameter. *F* values having *p* < 0.05 were recognized as statistically significant. Moreover, in the case when the ANOVA/MANOVA analysis revealed an interactive effect of cadmium and AME, the possible character of the interaction was described based on the comparison of the effect of the co-administration of cadmium and AME to the sum of effects noted as a result of their separate administration. The effect of cadmium or/and AME was expressed as a percentage change or a factor of change in a measured parameter compared to the control group. Based on the obtained results it was estimated whether the interaction had an antagonistic (Cd + AME effect < Cd effect + AME effect), additive effect (Cd + AME effect = Cd effect + AME effect) or another character [23].

Spearman rank correlation analysis was carried out to estimate mutual dependences between the measured markers of the oxidative-reductive status of the submandibular gland, as well as between these parameters and cadmium concentration in this salivary gland. A correlation is considered statistically significant at a correlation coefficient (*r*) having *p* < 0.05. In the case of *r* having a negative value and *p* < 0.05, the correlation is recognized as negative, whereas, in the case of positive *r* value (and *p* < 0.05), the correlation is positive in character.

## 3. Results

### 3.1. Effect of AME on the Antioxidative Status of the Submandibular Gland of Rats Treated with Cadmium

**Figure 1:**
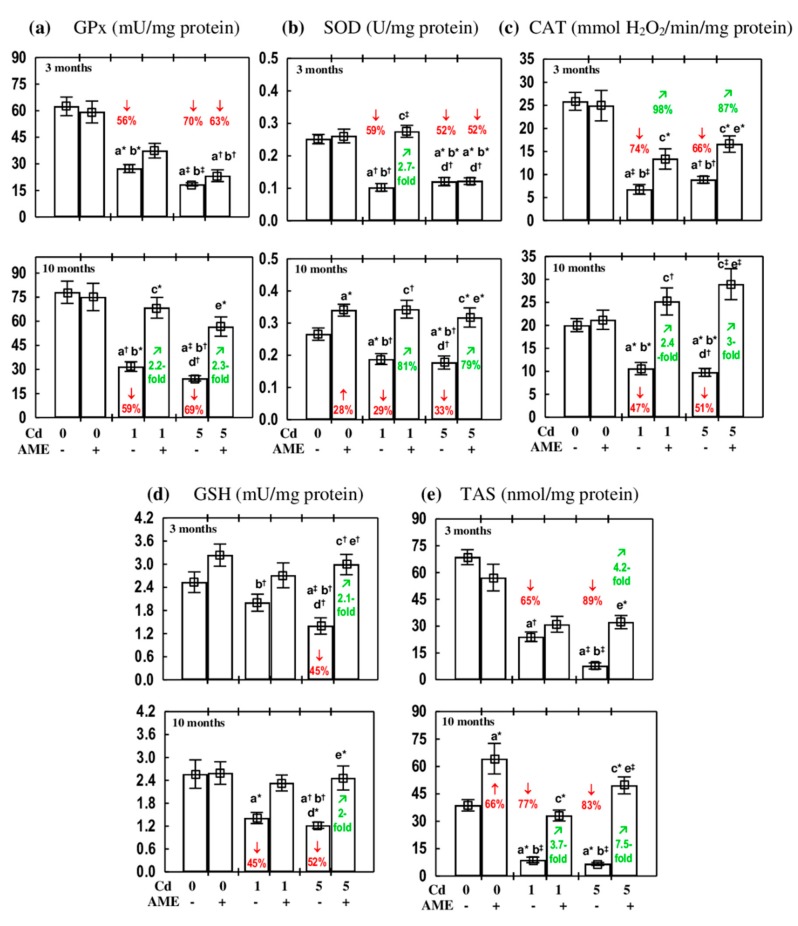
The effect of the extract from *Aronia melanocarpa* L. berries (AME) on the markers of the antioxidative status of the submandibular gland of female rats exposed to cadmium (Cd). (**a**) glutathione peroxidase (GPx) activity; (**b**) superoxide dismutase (SOD) activity; (**c**) catalase (CAT) activity; (**d**) reduced glutathione (GSH) concentration; (**e**) total antioxidative status (TAS). The animals received cadmium in the diet at concentrations of 0, 1, and 5 mg Cd/kg and/or 0.1% aqueous AME (+) or not (−). Data are presented as mean ± SE for eight rats. Statistically significant differences (Kruskal–Wallis post hoc test): ^a^ compared to the control group, ^b^ compared to the AME group, ^c^ compared to the Cd_1_ group, ^d^ compared to the Cd_1_ + AME group, ^e^ compared to the Cd_5_ group, where * p < 0.05, ^†^ *p* < 0.01, and ^‡^ *p* < 0.001. Numerical values in bars or above the bars reveal the percentage changes or factors of changes in comparison to the respective control group (↓, decrease; ↑, increase;) or the appropriate group that received cadmium alone (↗, increase).

The administration of AME alone for up to 10 months had no impact on the measured indices of the antioxidative status of the submandibular gland (GPx, SOD, CAT, GSH, and TAS) except for an increase in the activity of SOD and the value of TAS after 10 months (Figure 1).

In the rats maintained on the diets containing 1 and 5 mg Cd/kg for 3 and 10 months, the activities of antioxidative enzymes (GPx, SOD, and CAT) in the submandibular gland and TAS were decreased at each time point (by 29%-74% and 65%-89%, respectively; Figure 1). The concentration of GSH was also decreased (by 45%-52%); however, at the lower level of exposure this effect was noted only after 10 months (Figure 1).

The administration of AME under exposure to the 1 and 5 mg Cd/kg diet completely prevented these xenobiotic-induced changes in the determined markers of the antioxidative status of the submandibular gland, except for the decrease in the activities of GPx and SOD due to the 3 month feeding with the diet containing 5 mg Cd/kg. Apart from these two exceptions, the values of all determined indices of the antioxidative status in the animals receiving AME during the treatment with cadmium did not differ compared to the respective control group (Figure 1).

The ANOVA/MANOVA analysis revealed that the improvement in the antioxidative status of the submandibular gland due to the administration of AME to the rats treated with cadmium (1 and 5 mg Cd/kg diet) was the result of the independent action of the extract and/or its ingredients interaction with this heavy metal, which seemed to be antagonistic in character (Table 2). However, this analysis revealed the lack of an effect of the extract (both independent and interactive) on TAS in the Cd_1_ + AME group after 3 months, in spite of the fact that the value of this parameter in this group did not differ compared to the control group, whereas in the Cd_1_ group it was decreased by 65% (Figure 1).

### 3.2. Effect of AME on the Oxidative Status of the Submandibular Gland of Rats Treated with Cadmium

**Figure 2:**
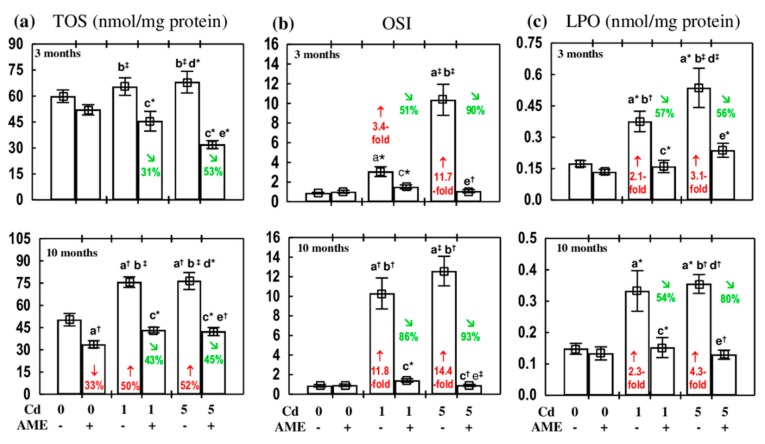
The effect of the extract from *Aronia melanocarpa* L. berries (AME) on the markers of oxidative status of the submandibular gland of female rats exposed to cadmium (Cd). (**a**) total oxidative status (TOS); (**b**) oxidative stress index (OSI); (**c**) lipid peroxides (LPO) concentration. The animals received cadmium in the diet at concentrations of 0, 1, and 5 mg Cd/kg and/or 0.1% aqueous AME (+) or not (−). Data are presented as mean ± SE for eight rats. Statistically significant differences (Kruskal–Wallis post hoc test): ^a^ compared to the control group, ^b^ compared to the AME group, ^c^ compared to the Cd_1_ group, ^d^ compared to the Cd_1_ + AME group, ^e^ compared to the Cd_5_ group, where * p < 0.05, ^†^ *p* < 0.01, and ^‡^ *p* < 0.001. Numerical values in bars or above the bars reveal the percentage changes or factors of changes in comparison to the respective control group (↓, decrease; ↑, increase;) or the appropriate group that received cadmium alone (↘, decrease).

The administration of AME alone for up to 10 months had no influence on the estimated markers of the oxidative status of the submandibular gland (TOS, OSI, and LPO), except for a decrease in TOS after 10 months (Figure 2). 

The 3 month treatment with the 1 and 5 mg Cd/kg diet had no influence on TOS of the submandibular gland; however, after 10 months of the experiment, the value of this parameter was higher (by 50% and 52%, respectively) compared to the control group (Figure 2). In all groups of the animals receiving AME under the exposure to cadmium, TOS was lower (by 31%–53%) than in the appropriate groups treated with this metal alone and did not differ compared to the respective control groups (Figure 2).

The exposure to the 1 and 5 mg Cd/kg diet resulted in an increase in OSI (3.4- to 14.4-fold) and the concentration of LPO (2.1- to 4.3-fold) in the submandibular gland, whereas the co-administration of AME entirely prevented these changes. The value of OSI and the concentration of LPO in all groups of rats co-administered with AME were within the ranges of values noted in the respective groups that did not receive the extract under the treatment with cadmium (Figure 2).

The two-way analysis of variance showed that the beneficial impact of the administration of AME under exposure to cadmium on the estimated markers of the oxidative status of the submandibular gland was the result of the independent impact of the extract and its interactive action with cadmium, which was antagonistic in character (Table 3).

### 3.3. Effect of AME on Cadmium Concentration in the Submandibular Gland of Rats Treated with this Heavy Metal

**Figure 3:**
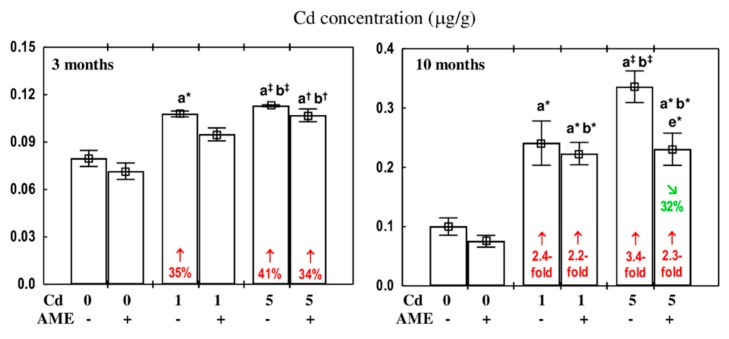
The effect of the extract from *Aronia melanocarpa* L. berries (AME) on cadmium (Cd) concentration in the submandibular gland of female rats exposed to this xenobiotic. The animals received cadmium in the diet at the concentration of 0, 1, and 5 mg Cd/kg and/or 0.1% aqueous AME (+) or not (−). Data are presented as mean ± SE for eight rats. Statistically significant differences (Kruskal–Wallis post hoc test): ^a^ compared to the control group, ^b^ compared to the AME group, ^e^ compared to the Cd_5_ group, where * p < 0.05, ^†^ *p* < 0.01, and ^‡^ *p* < 0.001. Numerical values in bars or above the bars reveal the percentage changes or factors of changes in comparison to the respective control group (↑, increase) or the appropriate group that received cadmium alone (↘, decrease).

The administration of AME alone throughout the experiment had no effect on the concentration of cadmium in the submandibular gland of rats (Figure 3). 

Cadmium concentration in the submandibular gland of the rats feed with the 1 and 5 mg/kg diet in each time point was higher (by 35% and 41% after 3 months and 2.4- and 3.4-fold after 10 months, respectively) than in the control animals (Figure 3).

In the rats administered with AME under the 3 month exposure to the 1 mg Cd/kg diet, the concentration of cadmium in the submandibular gland did not differ compared to the control group. However, after 10 months of the experiment this heavy metal concentration in the Cd_1_ + AME group was higher than in the control group (2.2-fold) and maintained within the range of the Cd_1_ group (Figure 3). In the case of the extract administration to the rats fed with the 5 mg Cd/kg diet, cadmium concentration in this salivary gland after 3 months did not differ compared to the Cd_5_ group and was 34% higher than in the control animals. After 10 months, the concentration of this toxic element in the Cd_5_ + AME group was lower (by 32%) than in the Cd_5_ group; however, it was higher (2.3-fold) than in the control animals (Figure 3).

The ANOVA/MANOVA analysis revealed that the impact of the co-administration of AME under the 3-month exposure to the 1 mg Cd/kg diet on cadmium concentration in the submandibular gland resulted from its independent (*F* = 17.64, *p* < 0.001) and interactive action with this xenobiotic (*F* = 8.393, *p* < 0.01). The clear protection against cadmium accumulation in this salivary gland due to the 10 month administration of AME at the treatment with the 5 mg Cd/kg diet was an effect of an independent action of this extract (*F* = 9.615, *p* < 0.01).

### 3.4. Mutual Relationships Between Investigated Markers of the Oxidative/Antioxidative Status of the Submandibular Gland and Cadmium Concentration in this Salivary Gland

Numerous mutual dependences were noted between the markers of the oxidative/antioxidative status of the submandibular gland (Table 4). Mutual positive correlations occurred between the particular indices of the antioxidative status (GPx, SOD, CAT, GSH, and TAS; Table 4). Similarly, mutual positive correlations were noted between the markers of oxidative status (TOS, OSI, and LPO; Table 4). Negative correlations occurred between particular indices of the antioxidative and oxidative status (Table 4).

Cadmium concentration in the submandibular gland negatively correlated with all markers of the antioxidative status of this salivary gland (GPx, CAT, GSH, and TAS), except for the activity of SOD, and positively with TOS, OSI, and the concentration of LPO (Table 4). 

## 4. Discussion

The present study is a part of a wide research project designed to investigate the possibility of using AME in protection from the unfavourable health outcomes of low-level and moderate chronic exposure to cadmium, and it provided new relevant and promising data in this regard. The study not only revealed that the extract improved the oxidative/antioxidative status when it was administered both alone and under exposure to cadmium, but it also allowed for better understanding of the effect of this toxic metal on the salivary glands.

In the available literature, some data showing that cadmium has a damaging impact on the salivary glands can be found [19,20,33,34,37,38]. Previously, we have reported the destruction of the oxidative/antioxidative balance and pathological changes in the morphological structure of the sublingual and/or submandibular salivary glands; however, these effects were investigated at higher levels of exposure to cadmium (5 and 50 mg Cd/L in drinking water) than in the present study [19,20,37,38]. The knowledge that this xenobiotic may lead to the development of oxidative stress and enhance lipid peroxidation in the submandibular gland after 3 month low-level exposure and its low concentration in this salivary gland (0.108 ± 0.002 µg/g) is an important result of this investigation. This finding suggests that submandibular gland seems to be very sensitive to the destruction of the oxidative/antioxidative balance due to exposure to cadmium. Regardless of cause, oxidative stress has numerous negative consequences at the cellular level. By inducing conformative changes in all cell components, ROS triggers a number of disruptions in the morphological structure and damage to the physiological functions of cells, tissues, and organs. These frequently include irreversible oxidative damage to proteins, peroxidation of lipids and cell membranes, and modifications of bases of the deoxyribonucleic acid (DNA) and ribonucleic acid (RNA) [6,22,23]. Ultrastructural changes in the cells of the submandibular gland, such as a blurring of the structure of mitochondrial cristae, damage to the mitochondrial membranes, radiolucent lesions in the mitochondrial matrix, changes in the outline of cell nuclei, and chromatin lumping, have been reported by us in rats due to relatively high (50 mg Cd/L) chronic exposure to cadmium, but after the treatment with 5 mg Cd/L the ultrastructural picture was proper [37]. 

The factors of increase in the values of TOS in the submandibular gland tissue of the animals exposed to cadmium, compared to the respective controls, show that the extent of the intensity of this xenobiotic-induced oxidative stress depended on the level of exposure, and that at the low-level treatment it markedly intensified with its duration. The intoxication with cadmium first weakened the enzymatic antioxidative barrier (GPx, SOD, and CAT), but also decreased the concentration of the main non-enzymatic antioxidants, such as GSH, leading, as a result, to the decrease in TAS. Detailed analysis of changes in particular markers of the antioxidative status, together with the fact that the value of TOS in the submandibular gland at both levels of exposure to cadmium for 3 months did not differ compared to the proper value, allow for the conclusion that the primary mechanism of the pro-oxidative action of this xenobiotic on this salivary gland consists of weakening the antioxidative protection. A key role in preventing oxidative stress in living organisms is played by antioxidative enzymes such as GPx, glutathione reductase (GR), SOD, and CAT [6]. The cadmium-induced insufficiency of the antioxidative mechanisms resulted in the decrease in TOS leading to the destruction of the oxidative/antioxidative balance and the development of oxidative stress with its negative consequences, such as enhanced lipid peroxidation. Because the mechanisms of the pro-oxidative action of cadmium at the cellular level are widely reported [3,5,14,21,22,23], they were not described in detail in this article.

It is important to underline that Gonzalez et al. [39] have suggested a potential use of the determination of cadmium concentration in the saliva as a marker of exposure to this xenobiotic. These authors have reported that, in the case of inhabitants of areas polluted with cadmium, the concentration of this element in the saliva (0.25 μg/100 cm^3^) may be higher than in the blood and urine. Abdollahi et al. [33] have noted an inhibitory effect of cadmium on the excretory function of the salivary gland in rats. Moreover, 2 hours after the intraperitoneal administration to rats of 10 mg Cd/kg b.w., the authors observed almost a 2-fold decrease in TAS with a simultaneous decrease in the concentration of total thiol groups (–SH groups) and a 3-fold increase in the concentration of substances reacting with thiobarbituric acid (a marker of lipid peroxidation) in the submandibular saliva. These changes in the oxidative-reductive status of the saliva may reflect the destruction of the oxidative/antioxidative balance in the glands secreting the saliva, whereas the inhibition of the proper excretory function of these glands may facilitate cadmium accumulation in them.

The results of the current study regarding the submandibular gland, together with our more recent findings on the parotid gland [34], show that the former salivary gland, in spite of its lower ability to accumulate cadmium, is more susceptible to the pro-oxidative action of this xenobiotic. Cadmium concentration in the parotid gland after the 3 month treatment with the diet containing 1 mg Cd/kg reached 0.405 ± 0.029 µg/g and was not accompanied by the occurrence of oxidative stress (evaluated based on the value of OSI). Only after 10 months of low-level exposure oxidative stress in the parotid gland was noted, and it took place at markedly higher cadmium concentrations in this salivary gland tissue (0.733 ± 0.042 µg/g). Based on these findings, it can be concluded that the ability of salivary glands to accumulate cadmium and their susceptibility to damage by these xenobiotics differs depending on the kind of the salivary gland.

Our results on the impact of cadmium on the submandibular gland confirm that cadmium may be dangerous for health even at a low exposure and, at the same time, it enhances the significance of the present study focused on the protective impact of AME regarding the outcomes of cadmium action and the importance of the findings of the study. Moreover, the study allowed us to evaluate the influence of aronia extract on the oxidative-reductive status, not only under the low-level and moderate repeated exposure, but also at very low exposure resulting from the trace, but unavoidable, presence of this xenobiotic in the standard diet (0.0584 ± 0.0049 mg/kg diet in our study [30]). Detailed analysis of the results of the determination of the biomarkers of the antioxidative and oxidative status has revealed that prolonged administration of the extract at a daily dose of 51.7–104.7 mg/kg b.w. had beneficial impact both when it was used alone and in the case of intoxication with cadmium. The increase in the activity of SOD and the value of TAS, with a simultaneous decrease in TOS, noted after the 10-month administration of AME alone confirm the antioxidative properties of the extract.

The most important finding of the present study is revealing that the administration of AME under exposure to cadmium almost completely prevented the development of oxidative stress and enhanced lipid peroxidation in the submandibular gland. In the available literature there are few data on the impact of some polyphenolic compounds (curcumin, epigallocatechin gallate, and resveratrol) or polyphenol-rich products (green and black tea) on the oxidative-reductive status of the salivary glands and saliva [40,41,42]. Narotzki et al. [41] have suggested that drinking green tea may protect the epithelium of the oral cavity from damage due to oxidative stress. Walvekar et al. [40] have reported that curcumin administered at a daily dose of 30 mg/kg b.w. per 30 days provided effective protection against 5% D-galactose-induced oxidative stress in the submandibular gland in mice; however, its longer (45 days) use led to a decrease in the activity of antioxidative enzymes due to the accumulation of curcumin. Moreover, it has been noted that resveratrol, administered at relatively high doses, can protect from irradiation-caused damage to the salivary gland in rats [42].

Detailed analysis of the results of the present study show that the administration of AME offered protection from cadmium-induced oxidative stress irrespective of the intensity of intoxication with this xenobiotic, and that the beneficial impact of the extract was accompanied by the improvement of the enzymatic and non-enzymatic antioxidative barrier. Taking into account the results of the ANOVA/MANOVA analysis, the beneficial influence of AME on the submandibular gland during cadmium intoxication may be explained by the direct impact of the chokeberry extract, as well as an indirect action related to interactions between the extract ingredients and this toxic heavy metal. The direct effect may be explained by the high antioxidative properties of the ingredients of aronia berries [24,27], reflected in the report in our previous paper [23], and the high ability of the 0.1% AME to scavenge 1,1-diphenyl-2-picrylhydrazyl radical (DPPH·) and to prevent cadmium-induced oxidative stress and its consequences in the liver and bone tissue [21,22,23]. It is important to underline that the beneficial influence of AME might be due to the presence in the extract of not only polyphenols, but also other ingredients with a known ability to counteract cadmium toxicity, such as essential microelements (iron, zinc, and selenium), vitamin C and vitamin E, fiber, pectin, β-carotene, and triterpens [14,24,25,26]. However, it seems that both the direct and indirect effects might be due to the high abundance of polyphenolic compounds. The mechanism of the interactive protective action of AME might result mainly from the extract ability of not only polyphenols, but also pectin and fiber, to chelate divalent cadmium ions (Cd^2+^) which in this way influences the body turnover of this element [26,30], including, as was revealed in the present study, its accumulation in the submandibular gland. We have already reported that the administration of AME to the animals maintained on the 1 and 5 mg Cd/kg diet decreased cadmium absorption from the gastrointestinal tract and increased its excretion, with urine leading, in this way, to lower the body burden of this xenobiotic, including its lower accumulation in the kidney, liver and skeleton [30]. The negative correlations between cadmium and the values of markers of antioxidative stress, and positive relationships with TOS, OSI, and LPO, show that the pro-oxidative action of cadmium on the submandibular gland intensifies with an increase in this heavy metal accumulation. Thus, the decreased concentration of this toxic metal in the submandibular gland tissue due to the administration of AME might be one of the causes of the protective impact of the extract, apart from its antioxidative properties. The fact that the administration of the chokeberry extract under the exposure to the 1 mg Cd/kg diet almost completely prevented oxidative stress and provided complete protection from lipid peroxidation in spite of only slightly decreased cadmium concentration in this salivary gland allowed us to recognize that the favourable effect of the extract at a low exposure to this heavy metal might be determined by its strong antioxidative potential. Detailed analysis of the results of the ANOVA/MANOVA analysis also shows that the impact of AME on the oxidative-reductive status of this salivary gland might be more determined by an independent action of the extract than its interaction with cadmium.

Because the beneficial effect of aronia extract on the oxidative/antioxidative status of the submandibular gland has been reported by us for the first time, wider discussion of the results is impossible. However, it is important to note that, in these animals, the protective impact of the administration of AME has also been revealed regarding the parotid gland [34]. Although this study has important practical implications, we are also aware of its limitations. The main limitation is the fact that all our data on the protective effect of AME come from a female rat model, and thus our results refer first of all to the female salivary glands. Thus, further research is needed to explain whether similar effects will also occur in males. Moreover, the strong potential of the extract to counteract against the pro-oxidative action of cadmium may suggest that the consumption of aronia products will also provide protection of the organism in the conditions of exposure to other pro-oxidants. 

In sum, the present investigation has revealed for the first time that even low-level repeated exposure to cadmium may weaken the enzymatic and non-enzymatic antioxidative barrier of the submandibular gland, leading to the destruction of the oxidative/antioxidative balance and development of oxidative stress and enhanced lipid peroxidation. The fact that cadmium may induce oxidative stress and lipid peroxidation in an experimental model of human exposure shows that even low intoxication with this xenobiotic may create a risk of damage to the salivary gland. However, the most important and practically useful finding of this study is revealing that administration of the AME, both alone and under exposure to cadmium, improves the oxidative/antioxidative status of this salivary gland. The extract administration under low-level and moderate exposure to cadmium almost completely prevented xenobiotic-induced oxidative stress and lipid peroxidation. 

The beneficial effect of AME may result from an independent antioxidative impact of its ingredients, as well as from their interactive action with cadmium, resulting in a decrease in its accumulation in this salivary gland. The findings of this study, together with our more recent results on the beneficial influence of AME on the parotid gland, show that the extract may offer protection against the unfavourable impact of cadmium on the organs of the oral cavity. Moreover, these results provide further evidence that products from the berries of *A. melanocarpa* may be effective in the protection of the organism under the conditions of exposure to cadmium.

## Figures and Tables

**Table 1:** The daily intake of the extract from the berries of *Aronia melanocarpa* L. (AME) and cadmium (Cd) in particular experimental groups.

Group	Daily Intake of AME ^1^ Mean ± SE (Range) [mg/kg b.w.]		Daily Intake of Cd ^2^ Mean ± SE (Range) [μg/kg b.w.]	
	3 months	10 months	3 months	10 months
Control	–	–	4.709 ± 0.067	2.716 ± 0.060
			(4.342–4.976)	(2.487–3.055)
AME	91.53 ± 1.12	62.29 ± 1.87	4.615 ± 0.065	2.935 ± 0.063
	(86.6–96.4)	(51.7–70.8)	(4.328–4.952)	(2.569–3.109)
Cd_1_	–	–	77.50 ± 1.264 ^‡^	43.85 ± 0.60 ^‡^
			(69.93–83.88)	(41.45–47.22)
Cd_1_ + AME	97.47 ± 1.45	60.92 ± 1.07	80.93 ± 1.06 ^‡^	46.90 ± 0.84 ^‡^
	(88.3–104.6)	(55.6–64.8)	(74.57–84.88)	(42.62–51.41)
Cd_5_	–	–	383.65 ± 4.44 ^‡^	235.78 ± 2.55 ^‡^
			(368.25–404.76)	(222.35–249.44)
Cd_5_ + AME	92.41 ± 1.30	65.14 ± 1.77	385.92 ± 4.91 ^‡^	239.66 ± 1.43 ^‡^
	(85.3–98.8)	(55.1–71.1)	(349.68–401.8)	(234.17–248.51)

^1^ Data represent the mean daily intake of AME and its range throughout the 3- and 10-month study. The intake of AME in the control group, Cd_1_ group, and Cd_5_ group was assumed to be 0. Data on the intake of polyphenolic compounds in the animals received AME have been presented in our previous reports from studies in this experimental model [30,32]. ^2^ Data represent the mean daily intake of cadmium and its range throughout the 3 and 10 month study. The intake of cadmium in the control group and AME group was calculated based on this metal concentration determined by us in the standard diet (0.0584 ± 0.0049 mg/kg) [30], while the intake of this xenobiotic in the groups exposed to cadmium was calculated based on its concentration in the diet, declared by the manufacturer (1 or 5 mg Cd/kg). ^‡^ *p* < 0.001 compared to the control group (nonparametric Kruskal–Wallis test).

**Table 2:** Estimation of the main and interactive effects of cadmium (Cd) and the extract from *Aronia melanocarpa* L. berries (AME) on the markers of the antioxidative status of the submandibular gland of female rats ^1,2^.

Parameter	Duration (Months)	1 mg Cd/kg Diet + AME				5 mg Cd/kg Diet + AME			
		Main Effect of Cd	Main Effect of AME	Interactive Effect of Cd and AME	Possible Character of Cd – AME Interaction	Main Effect of Cd	Main Effect of AME	Interactive Effect of Cd and AME	Possible Character of Cd – AME Interaction
**GPx**	3	37.21 ^‡^	NS	NS	No interaction	83.10 ^‡^	NS	NS	No interaction
	10	16.38 ^‡^	6.638 *	9.123 ^†^	Antagonistic action 0 vs. −59 ^3^ + 0; 0 vs. −59	32.60 ^‡^	5.375 *	7.721 ^†^	Antagonistic action 0 vs. −69 + 0; 0 vs. −69
**SOD**	3	16.05 ^‡^	29.37 ^‡^	23.49 ^‡^	Antagonistic action 0 vs. −59 + 0; 0 vs. −59	79.68 ^‡^	NS	NS	No interaction
	10	NS	30.25 ^‡^	NS	No interaction	6.183 *	22.98 ^‡^	NS	No interaction
**CAT**	3	45.19 ^‡^	NS	NS	No interaction	34.72 ^‡^	NS	NS	No interaction
	10	NS	14.75 ^‡^	10.71 ^†^	Antagonistic action 0 vs. −47 + 0; 0 vs. −47	NS	22.27 ^‡^	17.46 ^‡^	Antagonistic action 0 vs. −51 + 0; 0 vs. −51
**GSH**	3	-	-	-	-	7.050 *	19.59 ^‡^	NS	No interaction
	10	6.835 *	NS	NS	No interaction	6.522 *	4.839 *	4.442 *	Antagonistic action 0 vs. −52 + 0; 0 vs. −52
**TAS**	3	49.65 ^‡^	NS	NS	No interaction	81.55 ^‡^	NS	14.32 ^‡^	Antagonistic action 0 vs. −89 + 0; 0 vs. −89
	10	40.84 ^‡^	27.21 ^‡^	NS	No interaction	21.37 ^‡^	46.00 ^‡^	NS	No interaction

^1^ The results of the two-way analysis of variance are presented as *F* values and the level of statistical significance (*p*). *F* values having *p* < 0.05 were recognized statistically significant (* *p* < 0.05, ^†^ *p* < 0.01, ^‡^ *p* < 0.001). NS – not statistically significant. ^2^ To estimate the possible character of the interaction between Cd and AME, the effect noted at their co-administration was compared to the sum of the effects after their separate administration (Cd + AME effect vs. Cd effect +AME effect). Cd effect, AE effect, and Cd + AME effect are expressed as percentage changes (-, decrease) of a measured parameter in comparison to the respective control group. ^3^ The values represent percentage changes. GPx, glutathione peroxidase; SOD, superoxide dismutase; CAT, catalase; GSH, reduced glutathione; TAS, total antioxidative status.

**Table 3:** Estimation of the main and interactive effects of cadmium (Cd) and the extract from *Aronia melanocarpa* L. berries (AME) on the indices of the oxidative status of the submandibular gland of female rats ^1,2^.

Parameter	Duration (Months)	1 mg Cd/kg Diet + AME				5 mg Cd/kg Diet + AME			
		Main Effect of Cd	Main Effect of AME	Interactive Effect of Cd and AME	Possible Character of Cd – AME Interaction	Main Effect of Cd	Main Effect of AME	Interactive Effect of Cd and AME	Possible Character of Cd – AME Interaction
**TOS**	3	NS	9.677 ^†^	NS	No interaction	NS	28.73 ^‡^	11.90 ^†^	0 = 0 + 0
	10	31.19 ^‡^	61.23 ^‡^	6.240 *	Antagonistic action	18.93 ^‡^	40.44 ^‡^	4.776 *	Antagonistic action
					0 vs. +50 ^3^ + (-33); 0 vs. +17				0 vs. +52 + (-33); 0 vs. +19
**OSI**	3	22.49 ^‡^	6.320 *	9.050 ^†^	Antagonistic action	35.79 ^‡^	32.95 ^‡^	34.98 ^‡^	Antagonistic action
					0 vs. +3.4-fold + 0;				0 vs. +11.7-fold + 0;
					0 vs. +3.4-fold				0 vs. +11.7-fold
	10	38.34 ^‡^	30.50 ^‡^	31.27 ^‡^	Antagonistic action	59.22 ^‡^	58.41 ^‡^	59.53 ^‡^	Antagonistic action
					0 vs. +11.8-fold + 0;				0 vs. +14.4-fold + 0;
					0 vs. +11.8-fold				0 vs. +14.4-fold
**LPO**	3	14.09 ^‡^	17.64 ^‡^	8.392 ^†^	Antagonistic action	21.04 ^‡^	11.11 ^†^	6.535 *	Antagonistic action
					0 vs. +2.1-fold + 0;				0 vs. +3.1-fold + 0;
					0 vs. +2.1-fold				0 vs. +3.1-fold
	10	6.973 *	6.438 *	4.636 *	Antagonistic action	105.8 ^‡^	122.6 ^‡^	109.2 ^‡^	Antagonistic action
					0 vs. +2.3-fold + 0;				0 vs. +4.3-fold + 0;
					0 vs. +2.3-fold				0 vs. +4.3-fold

^1^ The results of the two-way analysis of variance are presented as *F* values and the level of statistical significance (*p*). *F* values having *p* < 0.05 were recognized statistically significant (* *p* < 0.05, ^†^ *p* < 0.01, ^‡^ *p* < 0.001). NS—not statistically significant. ^2^ To estimate the possible character of the interaction between Cd and AME, the effect noted at their co-administration was compared to the sum of the effects after their separate administration (Cd + AME effect vs. Cd effect + AME effect). Cd effect, AME effect, and Cd + AME effect are expressed as percentage changes or factors of changes (-, decrease; +, increase) of a measured parameter in comparison to the respective control group.^3^ The values present percentage changes. TOS, total oxidative status; OSI, oxidative stress index; LPO, lipid peroxides.

**Table 4:** Mutual relationships between the investigated markers of the oxidative/antioxidative status of the submandibular gland and cadmium (Cd) concentration in this salivary gland of female rats.

Parameters		Markers of Antioxidative Status					Markers of Oxidative Status		
		GPx	SOD	CAT	GSH	TAS	TOS	OSI	LPO
Markers of antioxidative status	SOD	0.703 ^‡1^	-						
	CAT	0.632 ^‡^	0.570 ^‡^	-					
	GSH	0.431 ^‡^	0.297 ^†^	0.423 ^‡^	-				
	TAS	0.646 ^‡^	0.505 ^‡^	0.650 ^‡^	0.503 ^‡^	-			
Markers of oxidative status	TOS	−0.255 *	−0.329 ^†^	−0.357 ^‡^	−0.405 ^‡^	−0.445 ^‡^	-		
	OSI	−0.570 ^‡^	−0.491 ^‡^	−0.611 ^‡^	−0.486 ^‡^	−0.874 ^‡^	0.639 ^‡^	-	
	LPO	−0.587 ^‡^	−0.608 ^‡^	−0.573 ^‡^	−0.418 ^‡^	−0.602 ^‡^	0.525 ^‡^	0.646 ^‡^	^-^
Cd concentration		−0.299 ^‡^	NS	−0.277 *	−0.430 ^‡^	−0.529 ^‡^	0.223 *	0.472 ^‡^	0.275 ^†^

^1^ Data represent coefficients of correlation (*r*) and the level of statistical significance * p < 0.05, ^†^ *p* < 0.01, and ^‡^ *p* < 0.001. NS – a lack of correlation (*p* > 0.05). GPx, glutathione peroxidase; SOD, superoxide dismutase; CAT, catalase; GSH, reduced glutathione; TAS, total antioxidative status; TOS, total oxidative status; OSI, oxidative stress index; LPO, lipid peroxides.

## Abbreviations

AAS method	atomic absorption spectrometry
AME	extract from the berries of *Aronia melanocarpa* L.
ANOVA/MANOVA	a two-way analysis of variance
CAT	catalase
Cd	cadmium
CdCl_2_ × 2.5 H_2_O	cadmium chloride 2.5-hydrate
CV	coefficient of variation
FR	free radicals
GPx	glutathione peroxidase
GSH	reduced glutathione
kg b.w.	kg body weight
LPO	lipid peroxides
-OH group	hydroxyl group
OSI	oxidative stress index
p	level of statistical significance
r	correlation coefficient
ROS	reactive oxygen species
SD	standard deviation
SE	standard error
SOD	superoxide dismutase
-SH group	thiol group
TAS	total antioxidative status
TOS	total oxidative status
